# Osteonecrosis of the jaw as a possible rare side effect of annual bisphosphonate administration for osteoporosis: A case report

**DOI:** 10.1186/1752-1947-5-477

**Published:** 2011-09-23

**Authors:** Sven Otto, Karl Sotlar, Michael Ehrenfeld, Christoph Pautke

**Affiliations:** 1 Department of Oral and Maxillofacial Surgery, Ludwig-Maximilians-University, Lindwurmstraße 2a, 80337 Munich, Germany; 2 Department of Pathology, Ludwig-Maximilians-University, Thalkirchner Straße 36, 80337, Munich, Germany

## Abstract

**Introduction:**

Osteonecrosis of the jaw is a serious side effect in patients receiving nitrogen-containing bisphosphonates intravenously due to malignant diseases. Albeit far less frequently, osteonecrosis of the jaw has also been reported to occur due to the oral administration of nitrogen-containing bisphosphonates due to osteoporosis. Annual infusions of zoledronic acid have been recommended in order to improve patient compliance, to optimize therapeutic effects and to minimize side effects. To date, osteonecrosis of the jaw has not been linked to the annual administration of bisphosphonates.

**Case presentation:**

We report the case of a 65-year-old Caucasian woman suffering from osteoporosis who developed early stage osteonecrosis of the jaw in two locations, with chronic infections, after two months of oral bisphosphonate treatment and three annual administrations of zoledronic acid. Our patient was treated by fluorescence-guided resection of the necrotic jaw bone areas; local inflammation was treated by removal of a wisdom tooth and repeat root resections. Histopathology revealed typical hallmarks of osteonecrosis of the jaw.

**Conclusion:**

Osteonecrosis of the jaw may occur as a consequence of annual administrations of zoledronic acid. It is conceivable that, due to the pharmacological properties of bisphosphonates, a jaw bone that encounters frequent local inflammations is more likely to develop osteonecrosis.

## Introduction

Osteoporosis can be managed effectively with bisphosphonates. These antiresorptive drugs significantly prevent skeletal complications, particularly fractures. Side effects of bisphosphonate therapy are rare but potentially serious as exemplified in the bisphosphonate-related osteonecrosis of the jaw (ONJ). First described in 2003, [1] ONJ is defined by the presence of transmucosal or transcutaneous jawbone exposure for at least eight weeks, a history of bisphosphonate administration, and the absence of any history of irradiation to the head and neck region [2]. Retrospective studies have identified a prevalence of up to 19% in patients that have received intravenous bisphosphonate applications due to cancer with bone metastasis [3]. In contrast ONJ is rare in osteoporosis patients receiving oral bisphosphonates, where the prevalence approximates 0.1% [4] (equivalent to 7.8% of all cases of bisphosphonate-related ONJ [5]).

Recent studies have revealed that annual intravenous administration of zoledronic acid decreases bone turnover and increases bone density in postmenopausal women with osteoporosis, thereby reducing the risk of vertebral, hip and other fractures. This bisphosphonate regime is generally well tolerated and has a favorable safety profile. Indeed, to date, no reports of bisphosphonate-related ONJ have emerged [6].

This case described in this report suggests that annual infusions of zoledronic acid may lead to bisphosphonate-related ONJ and offers further insights into the pathomechanisms of ONJ.

## Case presentation

A 65-year-old female Caucasian patient, suffering from intraoral purulent discharge in her left mandibular angle and the front of her left upper jaw, was referred to our hospital by her dentist. Her medical history revealed that she had suffered from postmenopausal osteoporosis, which was initially treated with two months of alendronate (70 mg once weekly) administered orally, followed by three annual infusions of zoledronic acid (5 mg intravenously). In addition, our patient was allergic to penicillin and was treated for diabetes mellitus type II with metformin.

**Figure 1:**
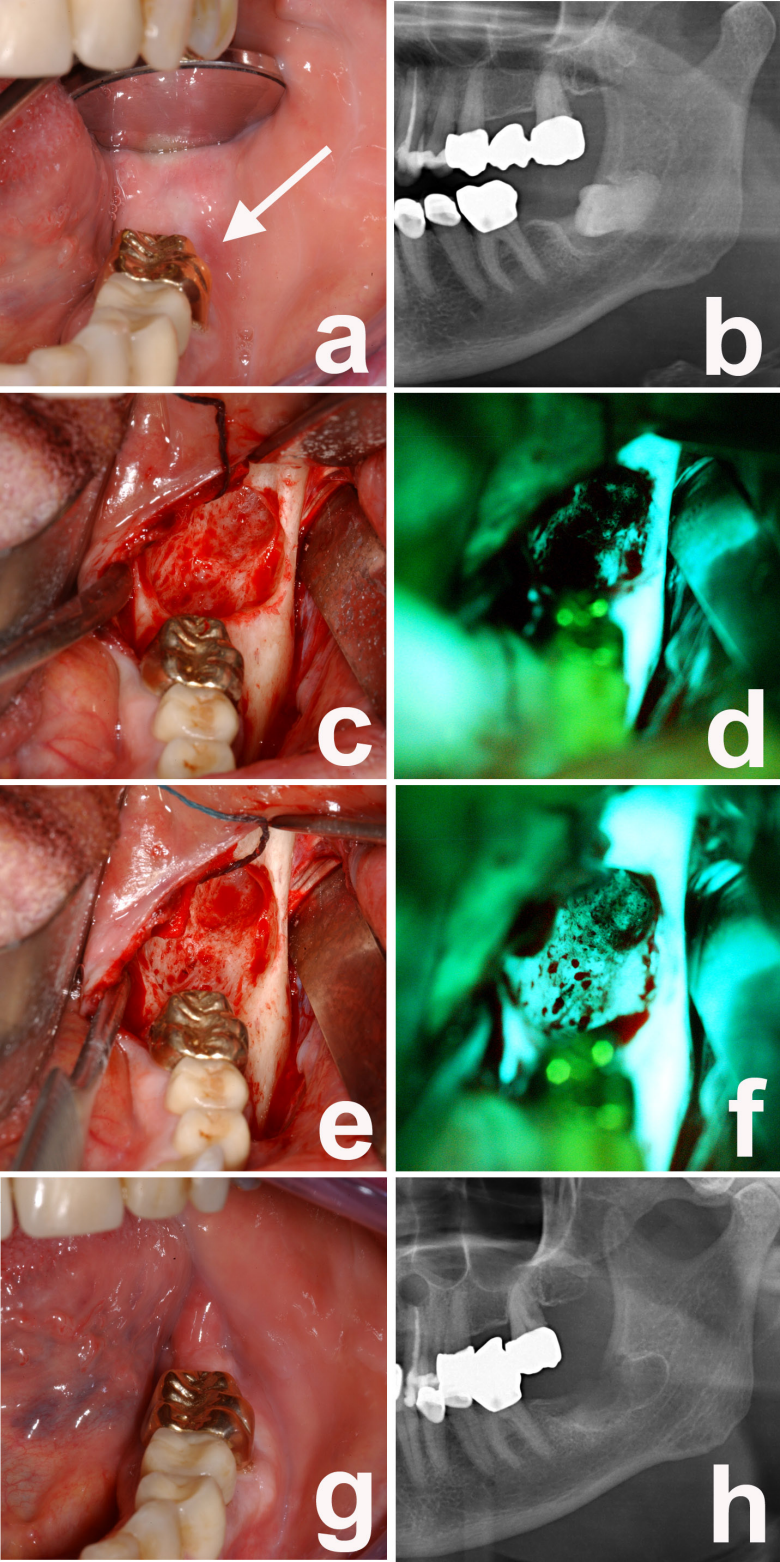
**a) Intraoral examination of her left upper jaw with fistula formation and pus on palpation in region 23; b) dental X-ray examination with gutta-percha pin in the fistula; c) intraoperative view with bony defect in region 23; d) fluorescence optic view with loss of fluorescence in region; e) bone cylinder region 23 with f) a mild fluorescence in the superficial areas and almost complete loss of fluorescence in deeper areas; g) corresponding clinical picture of the bone cylinder; h) histological examination of a representative biopsy with necrotic bone**.

**Figure 2:**
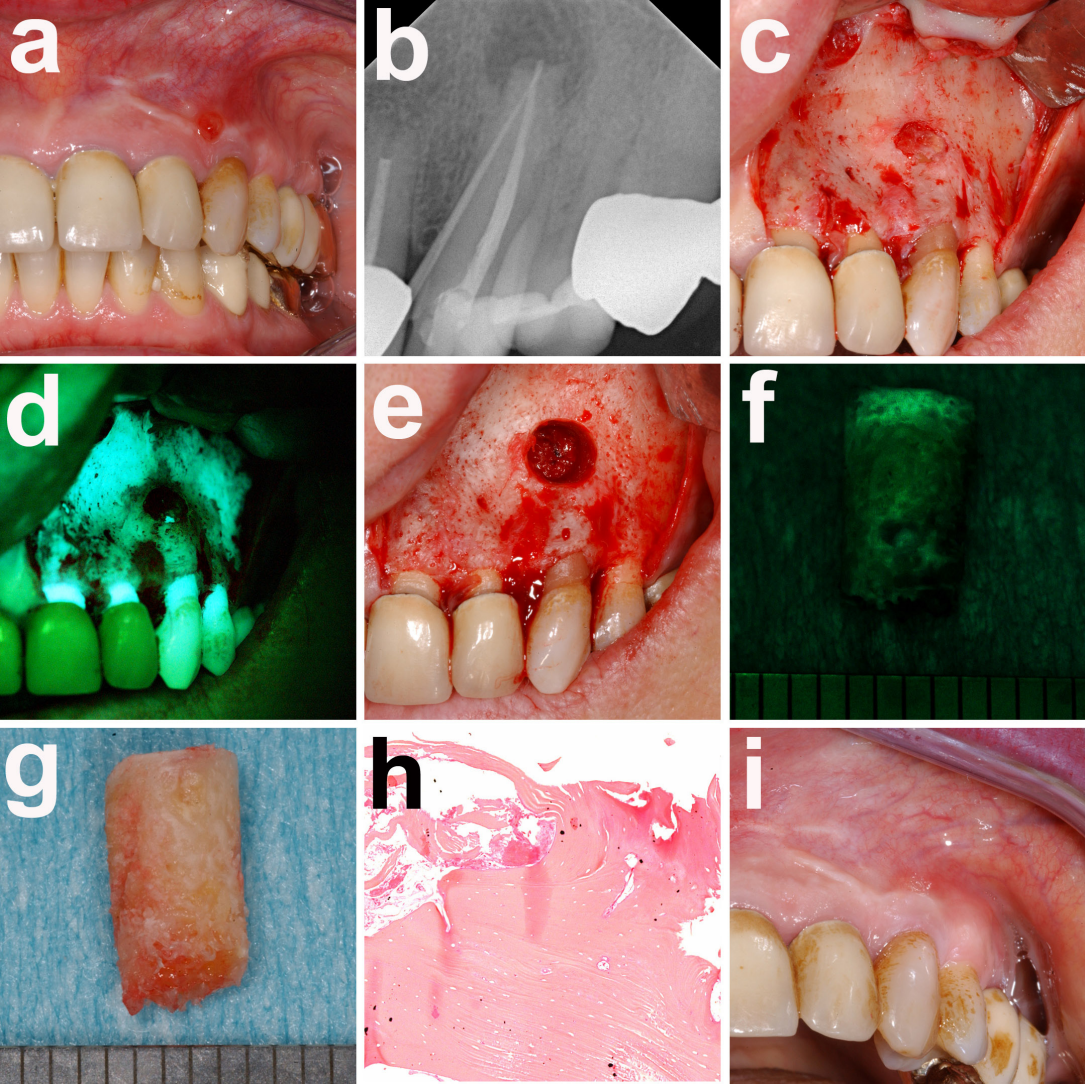
**a) intraoral examination of her left lower jaw with fistula formation and pus on palpation in region 38; b) panoramic radiograph with mixed radiopaque and radiolucent areas surrounding the retained wisdom tooth 38; c) intraoperative situs after wisdom tooth removal; d) corresponding fluorescence picture with loss of fluorescence in the lingual aspects of region 38; e) intraoperative situs after removal of necrotic bone parts; f) corresponding fluorescence picture with markedly enhanced fluorescence in the lingual aspects of region 38; g) intraoral examination eight weeks postoperatively with complete mucosal closure and without fistula formation; h) panoramic radiograph after removal of the wisdom tooth 38 and necrotic bone parts; i) intraoral examination eight weeks postoperatively with complete mucosal closure and without fistula formation**.

Intraoral examination revealed the presence of a fistula formation in her left mandibular angle in region 38 (Figure 1a) communicating with a retained left third molar. There was also a fistula formation in her upper jaw (region 22/23) communicating with her upper left lateral incisor and canine (teeth 22 and 23), which showed signs of chronic endodontic infections and had received endodontic and surgical treatment (root resection) in the past (Figure 2a). Both sites were marked by a purulent discharge on compression which was accompanied by mild to moderate pain on palpation. A panoramic radiograph and cone beam computed tomography identified radiolucent areas at the resected apices of teeth 22 and 23 as well as the region surrounding her left lower wisdom tooth (Figure 1b and 2b).

A fluorescence-guided removal of necrotic bone parts in her left mandibular angle was performed as previously described [7] and her left third molar was removed (Figure 1c-f). In addition, the upper jaw necrotic bone was resected under fluorescence-guidance and a root-resection of her lateral incisor and canine was carried out under general anesthesia (Figure 2c-g). Furthermore, her right third molar was removed. After the procedure, our patient received an intravenous antibiotic treatment (clindamycin 600 mg, three times daily) and was discharged five days later. The antibiotic was continued for 10 days at the same dose. During the follow-up there was no sign of infection and complete mucosal closure was achieved at all sites (Figure 1g and 2i).

Histological evaluation revealed the typical hallmarks of an early ONJ lesion, including areas of necrotic bone coinciding with signs of infections as well as areas with increased bone turnover (Figure 2h).

## Conclusion

Osteoporosis, a health threat of major public concern, is effectively managed with the oral administration of bisphosphonates. They significantly prevent skeletal complications, particularly fractures [8]. Although bisphosphonates are generally well tolerated and side effects are rare, bisphosphonate exposure has been linked to ONJ, which in recent years has been highlighted to potentially constitute a problem of serious clinical importance. ONJ is most prevalent in patients suffering from metastatic bone disease, who have received nitrogen-containing bisphosphonates intravenously. Cases of ONJ due to osteoporosis bisphosphonate therapy are less frequent [5].

Recent studies have proclaimed that the annual intravenous administration of zoledronic acid for osteoporosis therapy is safe, particularly regarding the development of ONJ [6]. In the HORIZON study, which encompassed 3876 patients, 76% (2950 patients) received three annual infusions of zoledronic acid and completed the follow-up [6]. Whilst no cases of ONJ were initially reported, database searches and expert adjudications identified two potential cases of ONJ (one in the placebo group and one in the zoledronic acid group). However, the reliability of ONJ diagnosis based on database searches or questionnaires (as frequently performed in retrospective studies) is questionable. Indeed, a recent study has suggested that the study design is of crucial importance and any retrospective study results in a significant underestimation of ONJ prevalence. It is certainly a drawback that the definition and diagnosis of bisphosphonate-related ONJ currently excludes histopathological evidence and relies predominantly on the medical history [2]. Since the inclusion of stadium 0 (no exposed bone, but unspecific symptoms of infection) in the staging of bisphosphonate-related ONJ [9], the diagnosis of early stages has to be considered to be vague, at best. Given that patients in stage 0 and I may only have unspecific symptoms (if any), it is of paramount importance to include detailed oral examinations in any diagnosis of bisphosphonate-related ONJ.

Despite the large number of patients included in the HORIZON trial, the follow-up period was relatively short (limited to 36 months following the commencement of the study and 12 months after the third and last infusion of zoledronic acid) [6]. In light of the fact that bisphosphonates have an extremely long half-life in bone, patients will not only continue to benefit but also remain at risk of developing bisphosphonate-related ONJ for an extended period, especially when an odontogenic infection is present or dentoalveolar surgical procedures are performed. Bisphosphonates bind to bone at around neutral pH and are released in acidic milieus. This physiologic mechanism takes place in the resorption lacunas during bone resorption, a feature that has been linked to the pathogenesis of ONJ [10]. Acidic conditions are common during infections and the jawbone is frequently subjected to acute and chronic infections. Indeed, in older patients (aged 65 or above) the prevalence of moderate to severe infections (periodontitis) exceeds 90%. The resulting change in pH may lead to a localized release and activation of bisphosphonates, which may trigger the onset of ONJ [10].

Detailed regular intraoral examinations are therefore imperative in order to treat dentoalveolar inflammations and detect early stages of ONJ lesions. If diagnosed timely, the outcomes of ONJ therapy are good; surgical approaches or conservative treatment strategies result in favorable outcomes in over 80% or 60%, respectively [7].

All patients receiving yearly infusions of bisphosphonates for osteoporosis should be adequately informed concerning the risk of ONJ. In addition, oral examinations and (where appropriate) preventive measures are called for in order to eliminate local inflammations--thereby minimizing the risk of ONJ manifestation.

## Consent

Written informed consent was obtained from the patient for publication of this case report and any accompanying images. A copy of the written consent is available for review by the Editor-in-Chief of this journal.

## Competing interests

The authors declare that they have no competing interests.

## Authors' contributions

SO, CP and ME analyzed and interpreted the patient data regarding the disease and wrote the manuscript. KS performed the histological examination and was a major contributor in writing the manuscript. All authors read and approved the final manuscript.
